# Genome-wide analysis of AGO, DCL and RDR gene families reveals RNA-directed DNA methylation is involved in fruit abscission in *Citrus sinensis*

**DOI:** 10.1186/s12870-019-1998-1

**Published:** 2019-09-12

**Authors:** Agustín Sabbione, Lucas Daurelio, Abelardo Vegetti, Manuel Talón, Francisco Tadeo, Marcela Dotto

**Affiliations:** 1 Facultad de Ciencias Agrarias, Universidad Nacional del Litoral, Esperanza, Argentina; 2 Laboratorio de Investigaciones en Fisiología y Biología Molecular Vegetal (LIFiBVe), Cátedra de Fisiología Vegetal, Facultad de Ciencias Agrarias, Universidad Nacional del Litoral, Esperanza, Argentina; 3 Consejo Nacional de Investigaciones Científicas y Técnicas (CONICET), Buenos Aires, Argentina; 4 Centre de Genómica, Institut Valencià d’Investigacions Agràries (IVIA), Montcada, València, Spain

**Keywords:** Small RNAs, DCL, AGO, RDR, Orange, Abscission, *Citrus sinensis*

## Abstract

**Background:**

Small RNAs regulate a wide variety of processes in plants, from organ development to both biotic and abiotic stress response. Being master regulators in genetic networks, their biogenesis and action is a fundamental aspect to characterize in order to understand plant growth and development. Three main gene families are critical components of RNA silencing: DICER-LIKE (DCL), ARGONAUTE (AGO) and RNA-DEPENDENT RNA POLYMERASE (RDR). Even though they have been characterized in other plant species, there is no information about these gene families in *Citrus sinensis,* one of the most important fruit species from both economical and nutritional reasons. While small RNAs have been implicated in the regulation of multiple aspects of plant growth and development, their role in the abscission process has not been characterized yet.

**Results:**

Using genome-wide analysis and a phylogenetic approach, we identified a total of 13 AGO, 5 DCL and 7 RDR genes. We characterized their expression patterns in root, leaf, flesh, peel and embryo samples using RNA-seq data. Moreover, we studied their role in fruit abscission through gene expression analysis in fruit rind compared to abscission zone from samples obtained by laser capture microdissection. Interestingly, we determined that the expression of several RNA silencing factors are down-regulated in fruit abscission zone, being particularly represented gene components of the RNA-dependent DNA Methylation pathway, indicating that repression of this process is necessary for fruit abscission to take place in *Citrus sinensis*.

**Conclusions:**

The members of these 3 families present characteristic conserved domains and distinct expression patterns. We provide a detailed analysis of the members of these families and improved the annotation of some of these genes based on RNA-seq data. Our data suggests that the RNA-dependent DNA Methylation pathway is involved in the important fruit abscission process in *C. sinensis.*

**Electronic supplementary material:**

The online version of this article (10.1186/s12870-019-1998-1) contains supplementary material, which is available to authorized users.

## Background

A wide variety of biological processes in plants are regulated by small RNAs, which are regulatory molecules of RNA, typically between 21 and 24 nucleotides long. In general, there are two main distinct small RNAs classes, which are known as microRNAs (miRNAs) and short-interfering RNAs (siRNAs). However, the vast number of siRNA sources described so far has rendered multiple subgroups in the siRNA class, including trans acting (ta-siRNAs), phased (pha-siRNAs), heterocromatic (hc-siRNAs) and natural antisense siRNAs (nat-siRNAs) among others [1, 2]. In plants, the biogenesis and action of these regulatory molecules depend mainly on members of the AGO, RDR and DCL families. RDRs are capable of synthesizing dsRNA using RNA as a template and DCLs are responsible for the cleavage of dsRNA into 21–24 nt long small RNAs thanks to their RNAseIII-type activity. In turn, the small RNA provide the specificity of action of the RISC complex containing the AGO factor, targeting at post-transcriptional level RNA molecules with partial or total base complementarity, which can be cleaved via the RNAseH-type activity of AGO proteins or through translation inhibition [3, 4]⁠. In plants, RDR proteins contain one unique conserved domain named RNA-dependent RNA polymerase (RdRP) [5, 6], whereas six domains are present in DCL proteins: DEXDc, Helicase-C, RNA-binding, PAZ, RNaseIIIa, RNaseIIIb (RIBOc) and Double Stranded RNA-binding (dsRB), but one or more may be missing [7]⁠. Within AGO proteins, there are four main domains, which are known as N-Terminal, PAZ, Mid and PIWI [8]⁠. Different studies have reported that plant DCL, RDR and AGO gene families are normally constituted by multiple members. A total of 20 genes coding for these protein families have been identified in Arabidopsis, 28 genes in tomato and maize [5, 9], 32 genes in rice [3]⁠ and 22 in grapevine and pepper [10, 11]⁠.

Sweet orange (*Citrus sinensis*) is one of the most important species for fruit consumption cultivated worldwide. Its nutritional attributes for human health are well known since it is an excellent source of easy access vitamin C [12], besides the immense economic importance of this species, for which a global production of US$9 billion was estimated in 2012 [13]⁠. From an agricultural point of view, abscission has a tremendous impact on yield, leading to high yield losses in key crops, including citrus species [14]. Small RNAs have been shown to participate in several aspects of plants growth and development, such as stress response, leaf polarity, flowering time [15–18]⁠ and resistance against diseases [19]. In the present work, we performed a genome-wide analysis in order to characterize these important protein families in *Citrus sinensis*. Using a phylogenetic approach, we identified and characterized the AGO, DCL and RDR gene families in orange and analyzed their expression patterns across five plant tissues, improving the gene structural annotation of five of them using RNA-seq data. Finally, we established that selected members of these families as well as additional single copy factors of the RNA-dependent DNA methylation (RdDM) pathway show differential expression in the fruit abscission zone of sweet orange samples analyzed using laser capture microdissection (LCM). A detailed analysis of the AGO, RDR and DCL gene families in *Citrus sinensis* is presented in this work and we provide initial evidence of an epigenetic component in the regulation of fruit abscission in this species.

## Results

### Identification and in silico analysis of AGO, RDR and DCL genes in orange

**Fig. 1:**
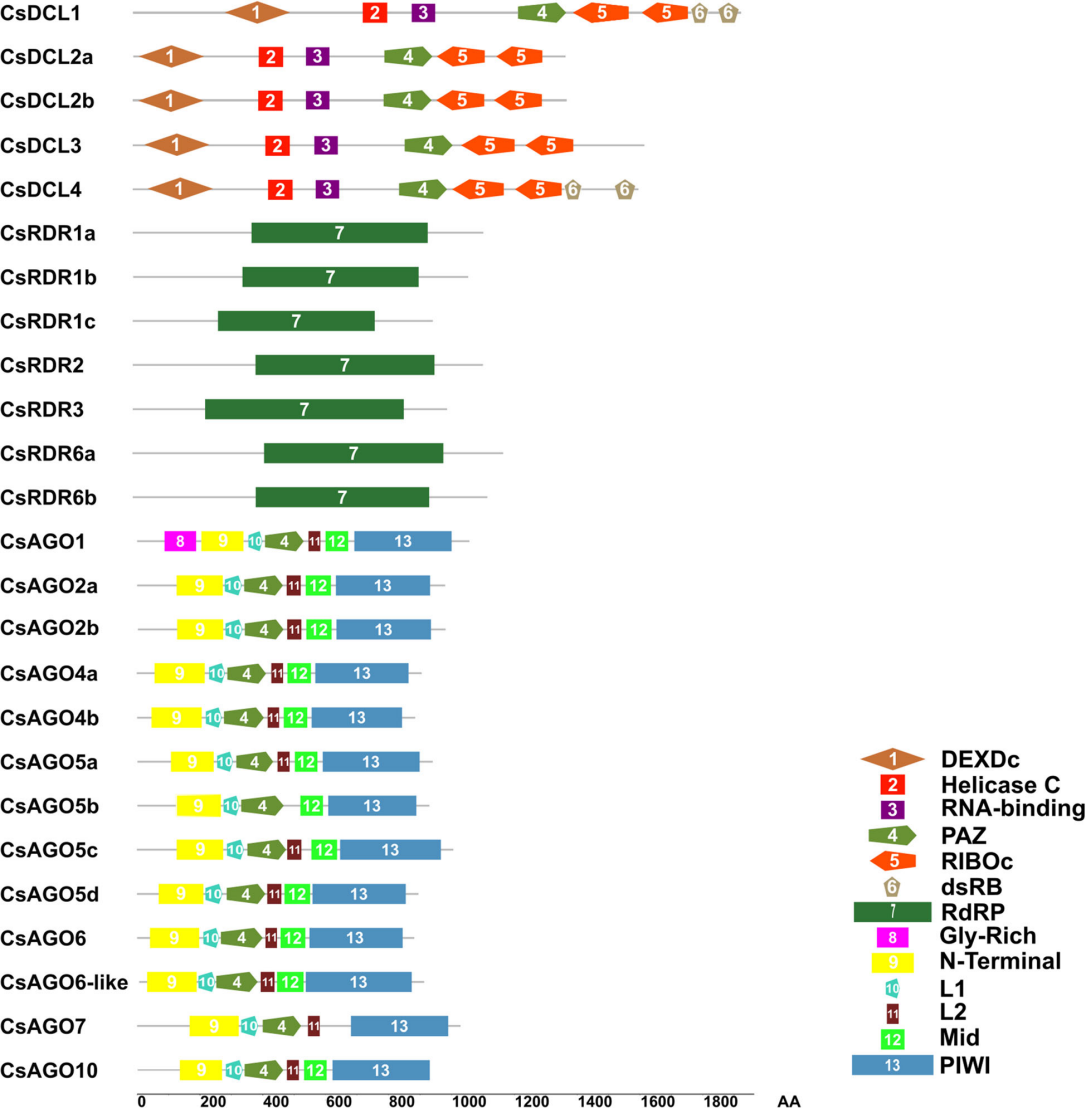
Domains present in AGO, DCL and RDR protein families from *C. sinensis*. 1: DEXDc; 2: Helicase C; 3: RNA-binding; 4: PAZ; 5: RIBOc; 6: dsRB; 7: RdRP; 8: Gly-Rich; 9: N-terminal; 10: L1; 11: L2; 12: Mid; 13: PIWI. Representation is in amino acid (AA) scale

In order to identify the AGO, RDR and DCL gene families in orange, we gathered data from previously characterized genes in Arabidopsis, rice, poplar and tomato. (Additional file 1). Using Arabidopsis sequences as queries in BLAST analyses, we identified five genes encoding DCL proteins (CsDCLs), seven encoding RDR (CsRDRs) and thirteen encoding AGO proteins (CsAGOs) in Pythozome (www.phytozome.org) and in the *“Citrus sinensis annotation project” (CSAP) (*http://citrus.hzau.edu.cn/orange/*)* databases (Table 1). Even though most of these genes were present in both databases, several inconsistencies were detected both in their functional and structural annotation across these two genome versions, as well as poor gene structural annotation for some of them. In general, a better structural annotation consistent with RNA-seq data was observed in CSAP database, except for genes orange1.1g001771m (CsRDR3) and orange1.1g002204m (CsAGO5a), which are better annotated in Phytozome (Table 1). In order to work with the most accurate gene models, we improved the annotation of five genes using information from both databases, as well as RNA-seq data from different plant tissues (Table 1; Additional file 2). For example, the CSAP annotated gene model for CsAGO5c is missing the Mid and PIWI domains; but when we used RNAseq data to update the structural annotation of this gene, all the typical AGO domains are detected in the newly annotated gene model (Fig. 1, Additional file 2). The characteristics of all the genes identified in this study are detailed in Table 1, including gene IDs in both databases, ORF length, protein length, isoelectric point (IP) and molecular weight (Mw), while the gene structure analysis and the updated protein sequences for the corrected gene structural annotations are detailed in Additional file 2. Also, the Pfam and SMART ID for all the domains identified in the CsAGO, CsRDR and CsDCL proteins are detailed in Additional file 3 and their associated Gene Ontology terms are detailed in Additional file 4, which describe the associated biological functions of these genes.

**Table 1:** Characterization of AGO, DCL and RDR genes in *Citrus sinensis*

	Gene Name	Gene ID Phytozome	Gene ID CSAP	Location	Protein			
					ORF length (bp)	Length (AA)	PI	Mw (Da)
DCL	CsDCL1	orange1.1g000174m	orange1.1 t00584	ChrUn: 6828329–6,838,566 (− strand)	5892	1963	5.96	219,934
	CsDCL2a	orange1.1g000607m	Cs6g03520	Chr6: 3969936–3,982,992 (− strand)	4191	1396	7.65	158,488
	CsDCL2b	orange1.1g003062m	Cs6g03500	Chr6: 3910063–3,922,849 (− strand)	4206	1401	6.48	158,770
	CsDCL3	orange1.1g000379m	Cs4g06370	Chr4: 3914566–3,925,649 (+ strand)	4959	1652	7.95	184,339
	CsDCL4	orange1.1g000380m	Cs4g01340	Chr4: 255555–267,608 (+ strand)	4902	1633	6.47	183,256
RDR	CsRDR1a	orange1.1g002586m	Cs2g17570	Chr2: 14327852–14,333,995 (+ strand)	3399	1132	7.13	129,642
	CsRDR1b	orange1.1g003789m	Cs5g14110	Chr5: 11659292–11,663,430 (− strand)	3249	1082	6.04	122,608
	CsRDR1c	orange1.1g035741m	–	Scaffold00168–67,689 – 71,577 (− strand)	2910	969	8.58	110,376
	CsRDR2	orange1.1g001183m	Cs5g05170	Chr5: 3069393–3,074,020 (+ strand)	3396	1131	6.59	128,958
	CsRDR3^1^	orange1.1g001771m	Cs4g15260	Chr4: 14278137–14,289,339 (+ strand)	3048	1015	6.8	115,671
	CsRDR6a	orange1.1g041430m	Cs7g05350	Chr7: 2849984–2,855,672 (+ strand)	3594	1197	6.13	136,437
	CsRDR6b^2^	orange1.1g048783m	Cs1g14730	Chr1: 18022840–18,027,238 (+ strand)	3501	1166	6.01	132,490
AGO	CsAGO1	orange1.1g001466m	Cs5g16710	Chr5: 15851375–15,859,311 (− strand)	3222	1073	9.38	118,338
	CsAGO2a	orange1.1g012649m	Cs2g10760	Chr2: 8058870–8,063,724 (+ strand)	2946	981	9.29	110,071
	CsAGO2b	–	Cs2g10770	Chr2: 8075960–8,080,634 (+ strand)	2946	981	9.26	110,304
	CsAGO4a	orange1.1g002449m	Cs2g29070	Chr2: 28612071–28,619,504 (− strand)	2763	920	8.98	102,998
	CsAGO4b	orange1.1g002636m	Cs3g06860	Chr3: 9639528–9,647,482 (+ strand)	2697	898	9.43	100,950
	CsAGO5a^1^	orange1.1g002204m	Cs7g17940	Chr7: 13746350–13,751,613 (− strand)	2865	954	9.27	106,773
	CsAGO5b^2^	orange1.1g003630m	Cs7g17970	Chr7: 13774473–13,781,129 (− strand)	2847	948	9.50	105,299
	CsAGO5c^2^	–	Cs7g17930	Chr7: 13732122–13,738,824 (− strand)	3021	1006	9.28	112,004
	CsAGO5d	–	Cs6g14430	Chr6: 15643144–15,650,122 (− strand)	2688	895	9.23	99,978
	CsAGO6	orange1.1g002661m	Cs2g20520	Chr2: 17331361–17,339,897 (− strand)	2688	895	9.41	100,500
	CsAGO6-like^2^	orange1.1g048669m	Cs6g16080	Chr6: 17010270–17,016,001 (− strand)	2814	937	9.71	105,659
	CsAGO7	orange1.1g001684m	Cs7g03360	Chr7: 1453333–1,457,453 (− strand)	3093	1030	9.24	117,036
	CsAGO10	orange1.1g001954m	Cs9g07740	Chr9: 4853982–4,862,787 (− strand)	2979	992	9.34	111,516

^1^ Phytozome gene structural annotation used

^2^ Updated gene structural annotation

All seven RDR genes found in the orange genome encode proteins with the RdRP domain. The number of amino acids of the members of this family varies between 969 and 1197 (Table 1). We improved the annotation of the gene orange1.1g048783m/Cs1g14730 (CsRDR6b) using RNA-seq data from leaf, root, embryo, flesh and peel libraries (Table 1 and Additional files 2 and 5). We observed expression in all the libraries in the region annotated as intron 3 for this gene, therefore the gene structure was updated (Additional file 2). The region of the updated sequence codes for the RdDP domain of this protein, which is predicted correctly using SMART after this re-annotation (Fig. 1), while the previous version of the gene showed a truncated RdDP domain.

The CsDCL family consists of five members for which the coding sequences range between 4191 and 5892 bp and code for proteins between 1396 and 1963 amino acids (Table 1). Pfam and SMART analyses revealed that all CsDCLs showed six conserved domains: DEXDc, Helicase C, RNA-binding, PAZ and two consecutive RIBOc domains (RNaseIIIa and RNaseIIIb). Besides these, CsDCL1 and CsDCL4 presented two consecutive dsRB domains after the RIBOc domains (Fig. 1).

The thirteen identified AGO proteins have the N-terminal, L1, PAZ and PIWI domains, whereas the L2 domain is present in twelve of these proteins, but is missing in orange1.1g003630m/Cs7g17970 (CsAGO5b). Also, the Mid domain is present in all proteins, except in orange1.1g001684m/Cs7g03360 (CsAGO7) and orange1.1g001466m/Cs5g16710 (CsAGO1) is the only one starting with a Gly-Rich domain (Fig. 1).

In addition to the conserved domains present in these proteins, we also examined the presence of conserved motifs in these gene families. In Arabidopsis, AGO proteins have DDH/H or DDD/H motifs in the PIWI domain, which are responsible for their endonuclease activity [20–22]. We found the DDH/H motif in CsAGO1, CsAGO7 and CsAGO10, and the DDD/H motif was present in CsAGO2a (Additional file 6). We also identified DDH/P motifs for CsAGO4a and CsAGO4b; a DDH/S motif in CsAGO6 and DDY/H and DDY/P motifs for CsAGO5a and CsAGO5b, respectively. Finally, a D-H/T motif was observed in CsAGO6-like protein (Additional file 6), which is therefore likely to lack endonuclease activity.

We detected the conserved DECH motif in all the CsDCL proteins, which is characteristic of plant DCL proteins [23, 24] and the DLDGD motif, which is typically present in the catalytic domain of RDR proteins [6, 25], was detected in 5 out of 7 of the CsRDR proteins (Additional file 6).

### Phylogenetic analysis allows identification of DCL, RDR and AGO putative orthologues in orange

**Fig. 2:**
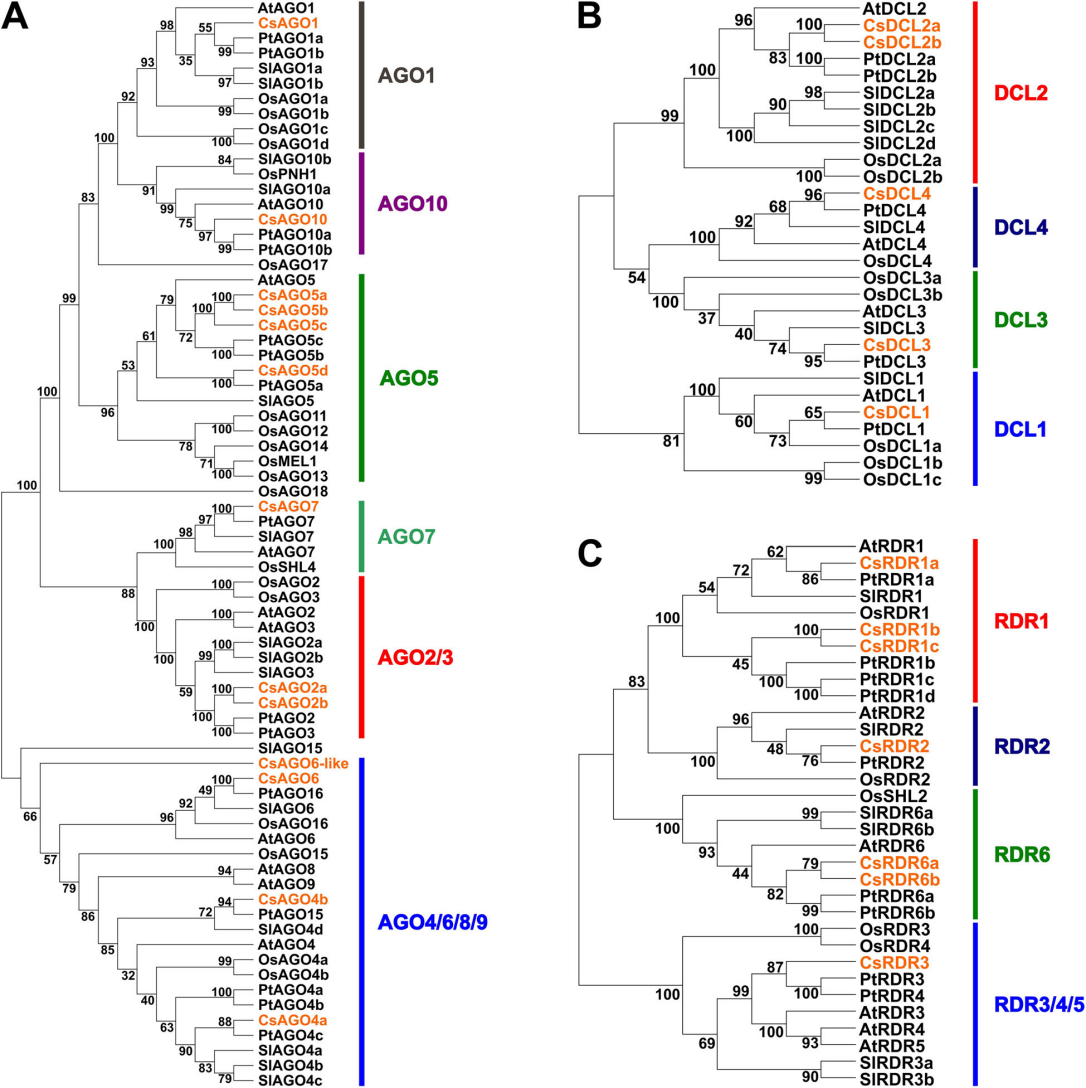
Phylogenetic analysis of DCL, AGO and RDR genes from orange, Arabidopsis, poplar, tomato and rice. Unrooted Neighbor-Joining trees were obtained from multiple alignments of (**a**) AGO, (**b**) DCL and (**c**) RDR protein sequences using Muscle algorithm in MEGA 6.0. Bootstrap values from 1000 replicates are indicated. Gene families are divided by clades, highlighted with different colors. *C. sinensis* genes are colored in orange

We used a phylogenetic approach to identify the putative orthologous genes from the AGO, RDR and DCL families in orange. We built an unrooted neighbor-joining tree for the three analyzed families using full length protein sequences from Arabidopsis, tomato, poplar and rice (Fig. 2). The AGO gene family was separated into six clades: AGO1, AGO10, AGO5, AGO7, AGO2/3 and AGO4/6/8/9 (Fig. 2a). We identified only one gene from the *C. sinensis* genome in each one of the clades containing AGO1, AGO10 and AGO7 members of the AGO family in the rest of the analyzed species, which we named CsAGO1, CsAGO10 and CsAGO7, respectively. Two AGO members were identified in the AGO2/3 clade and were named CsAGO2a and CsAGO2b, being the latter only present in the CSAP genome, sharing 93% amino acid identity with each other. Four *C. sinensis* proteins grouped in the AGO5 clade, sharing between 52 and 87% amino acid identity, and were named CsAGO5a, CsAGO5b, CsAGO5c and CsAGO5d, being the last two also only present in CSAP genome. Also four members of the orange AGO family grouped within the AGO4/6/8/9 clade; of these, we named two as CsAGO4a and CsAGO4b, while a third one was named CsAGO6, due to its proximity to AtAGO6 (Fig. 2a). Finally, one AGO protein in the AGO4/6/8/9 clade failed to group with other characterized proteins in this clade and was named CsAGO6-like due to its proximity to the AGO6 group (Table 1, Fig. 2a, for the corresponding gene IDs, please refer to Additional file 1 or Table 1).

The five CsDCL proteins were distributed in four clades: DCL1, DCL2, DCL3 and DCL4 (Fig. 2b). Two highly similar proteins with 90% of amino acid identity grouped in the DCL2 clade, and therefore were named CsDCL2a and CsDCL2b, respectively. The remaining three clades contain one CsDCL protein each, which we called CsDCL1, CsDCL3 and CsDCL4 accordingly (Table 1, Fig. 2b and Additional file 1).

Finally, the tree derived from CsRDRs sequences also consists of four clades: RDR1, RDR2, RDR6 and RDR3/4/5. The RDR1 clade contains three proteins: CsRDR1a, CsRDR1b and, only present in Phytozome genome, CsRDR1c (Table 1, Fig. 2c). Two proteins were placed in the RDR6 clade, CsRDR6a and CsRDR6b, both similar in length, with 1197 and 1145 amino acids, respectively and sharing 80% of their amino acid sequence. Finally, only one protein was located in each of the RDR2 and RDR3/4/5, which were named CsRDR2 and CsRDR3, respectively (Table 1, Fig. 2c, Additional file 1).

### Chromosomal location of orange DCL, RDR and AGO genes

**Fig. 3:**
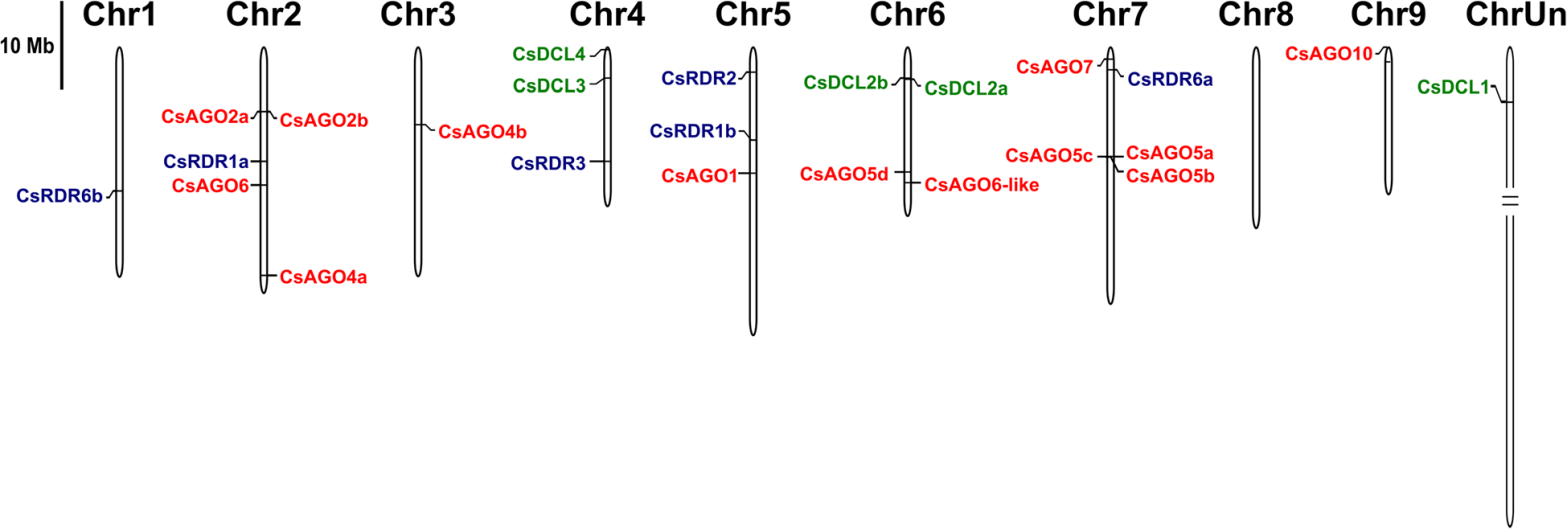
Genomic distribution of CsDCL, CsRDR and CsAGO genes. Chromosomes are represented on the scale indicated on the left of the figure. Different gene families are represented with different colors. 30 Mb were removed from “ChrUn” in order to fit the figure

Next, we analyzed the chromosomal distribution of all the DCL, RDR and AGO genes in sweet orange using CSAP database, which provides chromosomal location (Fig. 3). The genes corresponding to four CsDCL, five CsRDR and thirteen CsAGO proteins were unevenly located in all chromosomes, except for chromosome 8 which do not harbor any members of the analyzed families. Only one gene from the AGO family is encoded in each chromosome 3 and 9, namely CsAGO4b and CsAGO10, respectively. The paralogues CsDCL2a and CsDCL2b appear very close on chromosome 6, as well as CsAGO2a and CsAGO2b on chromosome 2 and CsAGO5a, CsAGO5b and CsAGO5c on chromosome 7 (Fig. 3, Table 1). Conversely, CsRDR1a and CsRDR1b are located in chromosomes 2 and 5 respectively. Chromosomes 2 and 7 contain five gene members of the analyzed families each, while chromosomes 4 and 5 host three of them. Only CsRDR1c was not annotated in this database, thus it is not represented in Fig. 3 (Table 1), while CsDCL1 is placed in the “Unknown chromosome” (ChrUn, Fig. 3 (Table 1), which contains sequence from the *C. sinensis* genome that could not be assigned to any of the chromosomes yet.

### Common and specific AGO, DCL and RDR gene expression patterns in different tissues

**Fig. 4:**
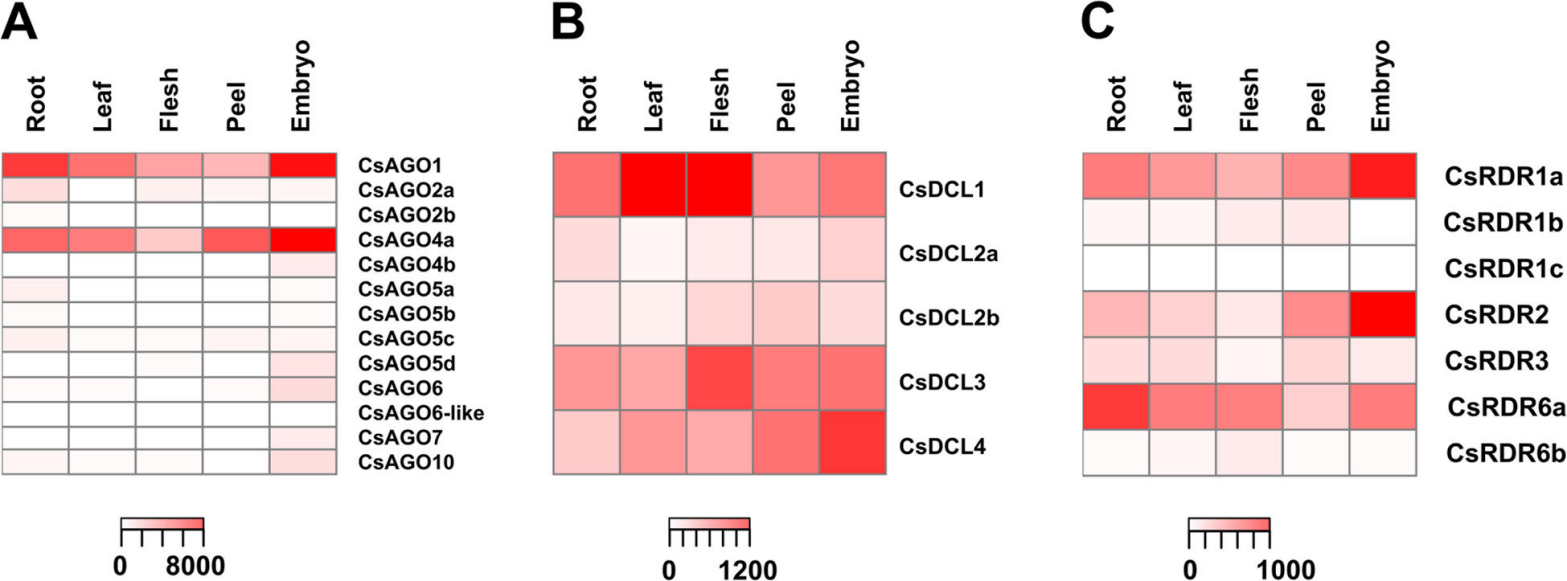
Expression levels of (**a**) CsAGO, (**b**) CsDCL and (**c**) CsRDR genes. Analyzed tissues were root, leaf, flesh, peel and embryo. Normalized expression for each gene family is represented with different color intensity, ranging from white to red

In order to characterize the expression pattern of the different members of these families, we analyzed RNA-seq data from five different tissues: leaf, root, flesh, peel and embryo (accession numbers for the raw data are detailed in Additional file 5). We performed differential expression analyses for all tissues (Additional file 7) and represented the expression levels using one heatmap plot per family (Fig. 4). CsAGO1 and CsAGO4a showed the highest expression levels within the AGO family and both accumulate to a much higher levels than the rest of the AGO genes analyzed. In order to better appreciate differences in expression, Additional file 8 shows the same heatmap plot from Fig. 4a without these two genes. Embryo tissue evidenced the highest expression for most of the AGO genes in comparison with the rest of the analyzed tissues, except for CsAGO2a, CsAGO2b, CsAGO5a and CsAGO5c, for which expression levels in roots are slightly higher (Fig. 4a; Additional file 8). Conversely, a weak expression of most genes is detected in flesh, peel and leaf, while CsAGO2b, CsAGO5b and CsAGO6-like presented very low expression levels in all tissues (Fig. 4a, Additional file 8).

All CsDCL proteins are expressed in the five analyzed tissues: CsDCL2a and CsDCL2b exhibit the lowest expression among this family, while CsDCL1 has the highest expression levels, especially in leaf and flesh. On the other hand, CsDCL3 and CsDCL4 show intermediate expression, presenting the highest accumulation in flesh and embryo, respectively (Fig. 4b).

Regarding the RDR family, CsRDR1c is not detected in any of the analyzed tissues (Fig. 4c, Additional file 7), CsRDR1b accumulates weakly in flesh, peel, leaf and root and CsRDR1a is the most abundant gene of this family across these samples. CsRDR2 is highly expressed in embryo and CsRDR6a accumulates in roots preferentially, whereas CsRDR6b is weakly expressed in all tissues and shows preferential accumulation in flesh (Fig. 4c, Additional file 7).

### Expression of genes involved in RdDM and ta-siRNA pathways show downregulation during orange fruit abscission

**Fig. 5:**
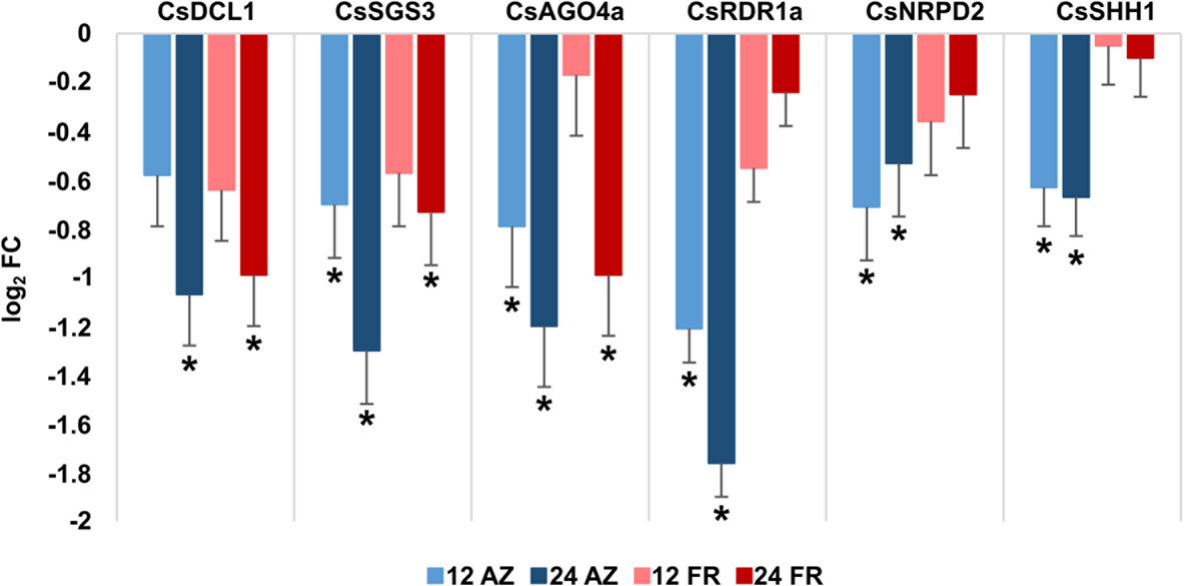
Relative expression of genes involved in RNA silencing. Comparisons between abscission zone (AZ) and fruit rind (FR) at 12 and 24 h after ethylene treatment. Values are represented relative to the expression in AZ or FR without ethylene treatment (0 h). Significant differential expression is indicated with an asterisk (* = q ≤ 0.05 and a log_2_ FC contrast cutoff value of ±0.5)

In order to gain insight into the role of RNA silencing pathways during the abscission process, we identified all the probes corresponding to small RNA-related factors present in the cDNA microarray used by [14], including the newly characterized AGO, RDR and DCL genes together with other genes coding for single-copy factors involved in the biogenesis and action of small RNAs (Fig. 5, Additional file 9). We analyzed expression data from abscission zone (AZ) in comparison to fruit rind (FR), obtained using LCM, after 12 or 24 h of ethylene treatment to induce abscission [14]. Interestingly, we detected downregulation in the expression of three members of the analyzed families: CsDCL1, CsRDR1a and CsAGO4a, as well as in three single-copy genes: the orthologue of SUPRESSOR OF GENE SILENCING 3 (CsSGS3), a component of the ta-siRNA biogenesis pathway; the orange orthologue of the subunit 2 of PolIV and Pol V (CsNRPD2) and the orthologue of SAWADEE HOMEODOMAIN HOMOLOGUE 1 (CsSHH1), which are involved in the RdDM pathway. Downregulation of these genes was also detected by qRT-PCR, except for CsRDR1 which was not tested using this method (Additional files 10 and 11). Of these, CsRDR1a, CsSHH1 and CsNRPD2 are specifically downregulated in the AZ and not in FR, both at 12 and 24 h after ethylene treatment (Fig. 5). CsSGS3 and CsAGO4a are downregulated specifically in AZ at 12 h, but at 24 h they are also downregulated in FR. Finally, CsDCL1 is downregulated both in AZ and FR after 24 h of ethylene treatment (Fig. 5). Some of these expression changes were also confirmed by qRT-PCR, and the overall tendency of downregulation of these genes was confirmed using this method (Additional file 10). These analyses show that a downregulation of several factors involved in small RNAs biogenesis and action is necessary during fruit abscission, which implies a still unexplored role for small RNAs in this important process.

## Discussion

RNA silencing is an ancient molecular mechanism which involves the participation of small RNAs acting at different levels in the regulation of gene expression. Plants have evolved a wide variety of pathways over different RDR-DCL-AGO combinations which have been shown to participate in several aspects of plant development [1, 2]. For example, DCL1 and AGO1 are mainly involved in the miRNA pathway, but no RDR protein is necessary for miRNA biogenesis [1, 26]. Some exceptional miRNA have evolved specialized modes of action, such as miR166 binding AGO10 exclusively to regulate shoot apical development and the also exclusive miR390/AGO7 combination in Arabidopsis to regulate AUXIN RESPONSE FACTORS 3 and 4 (ARF3 and ARF4) [27, 28]. RDR6, SGS3, AGO1 and DCL4 are the main components of the ta-siRNA pathway, responsible for the downregulation of target mRNAs at the post-transcriptional level in multiple plant species studied so far [29–31]; while RDR2, AGO4 and DCL3 participate in the RdDM pathway, mainly involved in the silencing of repetitive regions in the genome [32–34].

### RDR family in *Citrus sinensis* present distinctive features and member-specific expression patterns

RDRs are characterized by the presence of a conserved domain required to copy single-stranded RNA into double-stranded RNA, which was detected in all the members of this family identified in this work (Fig. 1). Also present in these proteins is a conserved DLDGD motif in the catalytic site, which was detected in 5 of the CsRDRs. Within this motif, lysine is often variable, and this was the case for CsRDR3, presenting a DFDGD motif, also detected in other species such as Arabidopsis, rice, pepper and coffee orthologues [6, 35, 36], whereas CsRDR1c presented a DQDGE motif, with two amino acid substitutions, which remains to be tested for functional activity (Additional file 6). The biological function of RDR proteins is usually linked to the subsequent action of specific DCL proteins and have been found in eukaryotic genomes including plants, fungi and invertebrate animals, but not in vertebrates and insects [37]. The different RDR family members exhibit functional diversification which is usually conserved across species; for example, RDR1 proteins have been shown to play a role in antiviral defense in Arabidopsis, pepper and potato [36, 38, 39]; RDR2 plays a critical role in RNA-directed DNA methylation and repressive chromatin modification which is conserved between Arabidopsis and maize, among other species [2, 32]. In addition to their participation in virus defense, RDR6 orthologues have been shown to participate in the ta-siRNA biogenesis pathway also in several species, having a key role in plant development through the regulation of the *TAS3* biogenesis pathway [29, 40]. In this study we identified seven members of the CsRDR family, including one RDR2 orthologue and one member grouping with the RDR3/4/5 clade (Fig. 3). Interestingly, we identified two RDR6 orthologues, which was also the case for tomato and other solanaceae like potato and *S. commersonii* [41], and three RDR1 orthologues, only seen in the citrus genome. This expansion in gene number was not observed in Arabidopsis, rice and tomato, which have only one member in the RDR1 clade (Fig. 2), whereas poplar (*P. trichocarpa*), a species that is evolutionary closer to orange, presents four RDR1 orthologues (Fig. 2) [42]. The members of this family showed distinct expression patterns in the tissues analyzed (Fig. 4), being CsRDR1a, CSRDR2 and CsRDR6a the most abundant. CsRDR1c was not detected in the analyzed samples, suggesting that this particular gene could be important in a different plant tissue, at different developmental stages not analyzed here, or that its expression is induced when the plant is growing in a particular environmental condition [11, 43, 44]. Is important to mention that CsRDR1c is only present in the *Citrus sinensis* genome published in Phytozome but not in CSAP database (Table 1), this could be due to the fact that these genomes are in early versions and discrepancies and incomplete gene structural annotations are expected. CsRDR1b, CsRDR3 and CsRDR6b exhibited low expression levels (Fig. 4), but they show tissue preferences, suggesting they could play roles under specific circumstances in plant growth and development or that their expression could be induced in response to specific environmental cues.

### DCL family members in *Citrus sinensis* present different expression patterns

DCL endonucleases process dsRNA into small RNA duplexes with 2-nt 3′ overhangs. DCL1 is capable of recognizing imperfect stem-loop substrates present in pre-miRNA transcripts, while the rest of the DCLs found in plants are responsible for the 21-, 22 and 24-nt siRNA production [45]. Plant DCL genes form a monophyletic group spawned after the plant-animal split but before the monocot-dicot divergence 150 million years ago [46]. All the genes identified in this study harbor the DEXDc, Helicase C, RNA binding and two tandem RIBOc domains, whereas CsDCL1 and CsDCL4 showed 2 additional dsRB domains (Fig. 1). In addition, all of them presented the characteristic DECH motif (Additional file 6). The *C. sinensis* genome presents one member of each DCL1, DCL3 and DCL4 clades, similar to Arabidopsis and tomato, but noticeably two members of the DCL2 clade, which is also the case for the OsDCL2 proteins. CsDCL2 proteins, which are presumed responsible for the generation of 22-nt siRNAs based on their Arabidopsis counterpart, appear to have suffered tandem duplication considering the close location of CsDCL2a and CsDCL2b on chromosome 6 and the 90% identity in their amino acid sequence (Figs. 2 and 3). The different expression patterns detected for these two genes suggest they acquired distinct expression regulation and could have preferential roles in specific tissues. CsDCL1 showed expression in all tissues analyzed, which is consistent with its putative role in miRNA biogenesis [1, 26]. Similarly, CsDCL3 showed highest expression in flesh and CsDCL4 in embryo, but they are abundantly expressed in all samples analyzed, also consistent with their ubiquitous roles in RdDM and ta-siRNA biogenesis, respectively [2].

### AGO family in *Citrus sinensis* underwent tandem duplication events during evolution

AGO proteins are the main RNA silencing effectors across kingdoms, since they possess the slicing activity required by the small RNA-mediated regulatory pathways. The AGO family expanded during plant evolution, from ancient unicellular or multicellular green algae (e.g *Micromonas pusilla* and *Volvox carteri*), where three or less AGO genes are present, to ten or more members in flowering plants. The expansion of the AGO family in plants suggests a functional diversification of AGO proteins presumably due to expanding small RNA-directed regulatory pathways [47]. Eukaryotic AGOs contain four main domains: a variable N-terminal domain and the highly conserved PAZ, MID, and PIWI domains [1]. In this study, we identified these domains in all the characterized AGO proteins, with the exception of CsAGO7 missing the Mid domain (Fig. 1), which has been implicated in the sorting of small RNAs into different AGOs [48]. AGO7 has been shown to bind miR390 exclusively in Arabidopsis [27], but the basis for this unique AGO7-miR390 association is not completely understood and remains to be studied in *C. sinensis*. We also analyzed the presence of the characteristic DDH/H and DDD/H motifs in these proteins, which are present in Arabidopsis AGO proteins and their function has been extensively studied, establishing they are required for the slicing activity of AGO proteins [21, 22, 49]. Some amino acid substitutions have been detected in other species such as tomato and Brassica species, including DDH/P, DDH/S, DDY/H and DDY/P motifs detected in the CsAGO proteins identified in this study [5, 25]. However, CsAGO6-like did not present this motif, suggesting that this member of the CsAGO family may lack slicing activity (Additional file 6).

We identified single CsAGO1, CsAGO6, CsAGO7 and CsAGO10, while two highly identical CsAGO2 alleles (93% amino acid identity) were detected, possibly originated by tandem duplication based on their close location on chromosome 2 (Fig. 3, Table 1). Similarly, three CsAGO5 paralogues were detected in the orange genome in close locations on chromosome 7, also suggesting duplication events to give rise to these alleles sharing more than 90% amino acid identity (Fig. 3, Table 1). CsAGO5a and CsAGO5c have slightly higher expression in roots, whereas CsAGO5b has low expression in all the samples analyzed, suggesting new allele functionalization after the duplication events (Fig. 4). CsAGO1 exhibits high accumulation in most tissues as expected for its putative role as effector of miRNA-mediated regulation. CsAGO2 has preferential expression in roots, while CsAGO4a, CsAGO5d, CsAGO6 and CsAGO10 accumulate mostly in embryo. This could be related to a demand for CsAGO4 and CsAGO6, involved in the RdDM pathway during embryo development, since this pathway is of special importance during this process and others, like gametogenesis and meiosis, where hc-siRNAs are essential to silence the repetitive regions of the genome [2]; as well as a putative role of CsAGO10 analogous to the demonstrated role of AtAGO10 in embryo development [50, 51]. Similar to CsAGO5b, very low expression was observed for CsAGO7 and CsAGO4b (Additional file 8; Fig, 4); which might indicate a preferential role of these gene products in other tissues and/or developmental stages and/or during exposure to specific environmental conditions.

Is interesting to note that co-expression of factors involved in the same regulatory pathways has been detected in our expression analysis. For example, for miRNA biogenesis and action both CsDCL1 and CsAGO1 are needed, and co-expression of these genes was detected (Fig. 4). Similarly, RdDM requires the coordinated action of CsRDR2, CsDCL3 and CsAGO4 and the ta-siRNA pathway requires CsRDR6, CsDCL4 and CsAGO1, all of which have been shown to be co-expressed in our expression analysis (Fig. 4). However, is also important to take into consideration that the biological function of the small RNAs generated by these pathways are typically necessary in most plant tissues during plant growth and development. For example, miRNAs are key regulators of several transcription factors involved in leaf and fruit development [26, 52–54], as well as *TAS3*-derived ta-siRNAs, which regulate auxin response through cleavage of ARF transcripts [29–31], and gene silencing at the transcriptional level which is also regulated by RNA silencing components [55–57]. Therefore, different combinations of these factors are usually ubiquitously active. Elucidation of how the different combinations of RNA silencing factors are needed during plant growth and development is key to understand how the expansion of gene members of the AGO, RDR and DCL families have contributed to their unique functionalization in different species. Is also necessary to gain insight into the subcellular localization of the different members of these families, which have been well characterized in Arabidopsis and tomato, for example, but very little is known in other species [58, 59].

### Small RNA pathways are downregulated during fruit abscission

In citrus, fruit abscission represents a high percentage of annual yield losses. It is well established that plant growth hormones are deeply involved in abscission and that among them, ethylene is thought to be its natural regulator [60]. This process occurs specifically in the abscission zone (AC) of fruits and other organs, through coordinated changes in gene expression and is accelerated by ethylene treatment [14, 61, 62]. Our gene expression analysis in orange fruit abscission was oriented to analyze changes in the expression of RNA silencing-related genes, in a model system used before to analyze the molecular mechanisms underlying the abscission process induced by ethylene treatment [14, 61, 62]. Therefore we compared the expression of AGO, RDR, DCL and additional single gene factors with homology to known RNAi factors in the abscission zone of fruits (Fig. 5, Additional file 10). Interestingly, we detected a general downregulation of RNAi factors. In particular, we detected lower levels of expression for CsAGO4a, CsSHH1 and CsNRPD2 in the AZ. These genes participate in the RdDM pathway, which is an important RNAi-mediated epigenetic pathway in plants. The RdDM pathway is involved in transcriptional silencing of transposons and repetitive sequences and relies on specialized transcriptional machinery that includes the plant-specific RNA polymerases Pol IV and Pol V. Pol IV transcripts are rapidly processed into dsRNAs by RDR2, and subsequently processed into 24 nucleotide siRNAs by DCL3 and exported to the cytoplasm. In the cytoplasm, they are mainly incorporated into AGO4 containing complexes and imported back to the nucleus to target nascent transcripts transcribed by Pol V at the same loci, leading to DNA methylation [2]. Gene silencing also involves chromatin remodelation, typically through methylation of histone H3. The role of SHH1 in *A. thaliana* is to recognize H3K9me2 (dimethylation of lysine 9 in histone H3), which recruits Pol IV and initiates siRNA biogenesis for the maintenance of gene silencing [63]. Besides the RdDM pathway directed by 24-nt siRNAs, work in Arabidopsis showed that 21-nt ta-siRNAs can also direct RdDM. Instead of being processed by DCL4 and loaded into AGO1 to target mRNA cleavage in trans, these ta-siRNAs are processed by DCL1 and loaded into AtAGO4 or AtAGO6, directing methylation of *TAS* loci [64]. In this regard, the observed downregulation of CsDCL1 and CsSGS3 in FR or AZ could also contribute to a general inhibition of DNA methylation during the abscission process, as a result of the downregulation of these components of the *TAS*-related RdDM pathway and ta-siRNA biogenesis. The roles of RDR1 and RDR6 are usually redundant when they are involved in anti-viral defense and both produce DCL4 substrates [39]. Therefore, a role of CsRDR1 can be proposed in the context of ta-siRNA biogenesis and/or the *TAS* loci-related RdDM during the abscission process, instead of CsRDR6 for which no differential expression was observed. These changes in the expression of RNA silencing factors could lead to modification in the expression of *TAS* genes and other genomic regions regulated by the RdDM and the ta-siRNAs biogenesis pathways, presumably involved in the cellular processes participating in fruit abscission, such as cell wall disassembly [14].

The present work gives rise to interesting questions about potential epigenetic regulation of the abscission process. Further studies comprising genome-wide analysis of DNA methylation in the abscission zone and changes in small RNA population during the abscission process, together with their regulated genes are very interesting experiments that will help elucidate the molecular mechanisms involving RNA silencing during this interesting and economically important aspect of citrus biology.

## Conclusions

In the present work we identified and characterized 13 AGO, 5 DCL and 7 RDR genes present in the orange genome, through a careful search and analysis of the 2 available *C. sinensis* genomes. Their expression patterns across five plant tissues indicate that most of these genes are ubiquitously expressed but they show distinct levels of expression in different plant tissues. We further improved the annotation of five of these genes using RNA-seq data. Finally, we established that selected members of these families as well as additional single copy factors of the RdDM pathway (CsSHH1, CsNRPD2 and CsAGO4a) show differential expression in the fruit abscission zone of sweet orange samples analyzed using LCM, providing initial evidence of an epigenetic component in the regulation of fruit abscission in this species.

## Methods

### Identification of candidate AGO, DCL and RDR genes in orange

*Citrus sinensis* genome assembly (v1.1) and protein sequences were downloaded from Phytozome v12.1 (https://phytozome.jgi.doe.gov) and CSAP V2 (http://citrus.hzau.edu.cn/orange/). Amino acids sequences of all the DCL, AGO and RDR genes of *Arabidopsis thaliana* were retrieved from TAIR (https://www.arabidopsis.org/) and were used to search for orange orthologous genes with Phytozome’s online BLASTP tool. Conserved domains in orange’s proteins were analyzed and annotated using the Simple Modular Architecture Research Tool (SMART, http://smart.embl-heidelberg.de/) and the Pfam database (31.0, https://pfam.xfam.org/). Protein’s isoelectric point and molecular weight were calculated using Compute pI/Mw (http://web.expasy.org/compute_pi/). *Gene structures for* Additional file *2* *were created using GSDS 2.0* [65]*.*

### Phylogenetic analysis and chromosomal localization

Candidate proteins from *Citrus sinensis* were aligned with AGO, RDR and DCL proteins from *Arabidopsis thaliana, Solanum lycopersicum and Oryza sativa* using MEGA 6.0 [66]⁠. For the estimation of a phylogenetic tree, we used the MUSCLE algorithm (gap open, − 2.9; gap extend, 0; hydrophobicity multiplier, 1.2; clustering method, UPGMB) ([67]. A phylogenetic tree was built for each protein family using the Neighbor-Joining method with the bootstrap test replicated 1000 times. A Chromosomal location image was made according to genome annotations from the *“Citrus sinensis* annotation project*”* (CSAP) (http://citrus.hzau.edu.cn/orange/), using the online tool MapGene2Chromosome V2 (*http://mg2c.iask.in/mg2c_v2.0/*).

### Expression analysis of DCL, AGO, and RDR genes in orange tissues

We used publicly available libraries of RNA-seq to analyze the expression levels of these genes in different plant tissues. Accession numbers are detailed in Additional file 3. Raw reads were processed for removal of non-coding RNA sequences present in the Rfam 13.0 database (http://rfam.xfam.org/), using Bowtie version 1.1.1, allowing 2 mismatches [68]. Clean reads were mapped to the *Citrus sinensis* reference genome from CSAP or from Phytozome in the case of CsRDR1c and CsRDR6b, which are not annotated in the genome version from CSAP. We used Hisat2 software, version 2.1.0 [69], and hisat2-build to create the corresponding indexed genomes. Next, we used featureCounts 1.5.3 [70] to obtain read counts for each gene, using gene models from CSAP (csi.gene.models.gff3) or from Phytozome for CsRDR1c and CsRDR6b (Csinensis_154_v1.1.gene.gff3). Differentially expressed genes were selected as those showing a 2-fold change in expression using DESeq2 version 1.20.0 [71]⁠ with an adjusted *p*-value < 0.05.

### Expression analysis of DCL, AGO and RDR orange genes during fruit abscission

For gene expression in the abscission zone, data obtained from [14] was used. In this work gene expression was analyzed in Washington Navel orange fruits after 0, 12 and 24 h of ethylene treatment, used to promote abscission. RNA samples from abscission zone and fruit rind cells were obtained by LCM. For gene expression analysis a cDNA microarray including 21.081 putative genes of citrus was utilized [14]. Genes with an adjusted *p*-value ≤0.05 and a fold change of ±2 were considered differentially expressed.

### Quantitative RT-PCR analysis

Abscission zone and fruit rind tissue was dissected from Salustiana orange fruits after 0, 12 and 24 h of ethylene treatment, as described in [14]. Total RNA was prepared using Quick-zol reagent (Kalium Technologies) following manufacturer’s instructions and treated with DNase I (Promega). cDNA from 1000 ng of RNA per sample was synthesized using EasyScript (Trans) according to manufacturer’s protocol. Gene-specific primers were designed (Additional file 11) for use with TransStart Tip Green qPCR SuperMix (Trans). The specificity of all amplification products was determined using dissociation curve analyses. Relative quantification values were calculated based on three biological and three technical replicates using the 2^-ΔCt^ method, using Ubiquitin (CsUBI) expression as normalization control. Efficiency correction was necessary just for CsSGS3 primers (which was calculated 88%), as described in [72].

## Additional files

Additional file 1: Gene ID for AGO, RDR and DCL gene families used in the phylogenetic analysis (XLSX 13 kb)

Additional file 2: Gene structural annotation and updated protein sequences (PDF 461 kb)

Additional file 3; Pfam and SMART domain IDs (XLSX 12 kb)

Additional file 4: GO term annotation of the analyzed genes (XLSX 13 kb)

Additional file 5: Accession numbers of RNA-seq libraries used in this study (XLSX 11 kb)

Additional file 6: Motifs detected in CsAGO, CsRDR and CsDCL proteins (XLSX 13 kb)

Additional file 7: Differential expression analysis of CsAGO, CsDCL and CsRDR genes (XLSX 10 kb)

Additional file 8: Heatmap plots for the AGO family without CsAGO1 and CsAGO4a (TIF 3434 kb)

Additional file 9: Microarray data of RNA silencing factors in AZ and FR from *C. sinensis* fruits (XLSX 13 kb)

Additional file 10: Relative expression levels of RNA silencing genes. Transcript levels determined by qRT-PCR (mean ± SE; *n* = 3) compared to the expression of each gene in AZ or FR without ethylene treatment (0 h) and using CsUBI as normalization control (* *p* < 0.05; ** *p*-value< 0.001) (TIF 5450 kb)

Additional file 11: Primers used for qRT-PCR (XLSX 11 kb)

## Data Availability

All data generated or analysed during this study are included in this published article and its additional files.

## Abbreviations

AGO: Argonaute
DCL: Dicer-like
RdDM: RNA-directed DNA Methylation
RDR: RNA-dependent RNA Polymerase
