# Supplementary material for: Genome-wide analysis of AGO, DCL and RDR gene families reveals RNA-directed DNA methylation is involved in fruit abscission in Citrus sinensis
Source: BMC Plant Biol. 2019 Sep 12;19:401. doi: 10.1186/s12870-019-1998-1 (PMC6739940; doi:10.1186/s12870-019-1998-1)
Supplement: Supplementary file 2 — Gene structural annotation and updated protein sequences (PDF 461 kb) [file 12870_2019_1998_MOESM2_ESM.pdf]

Gene structural models

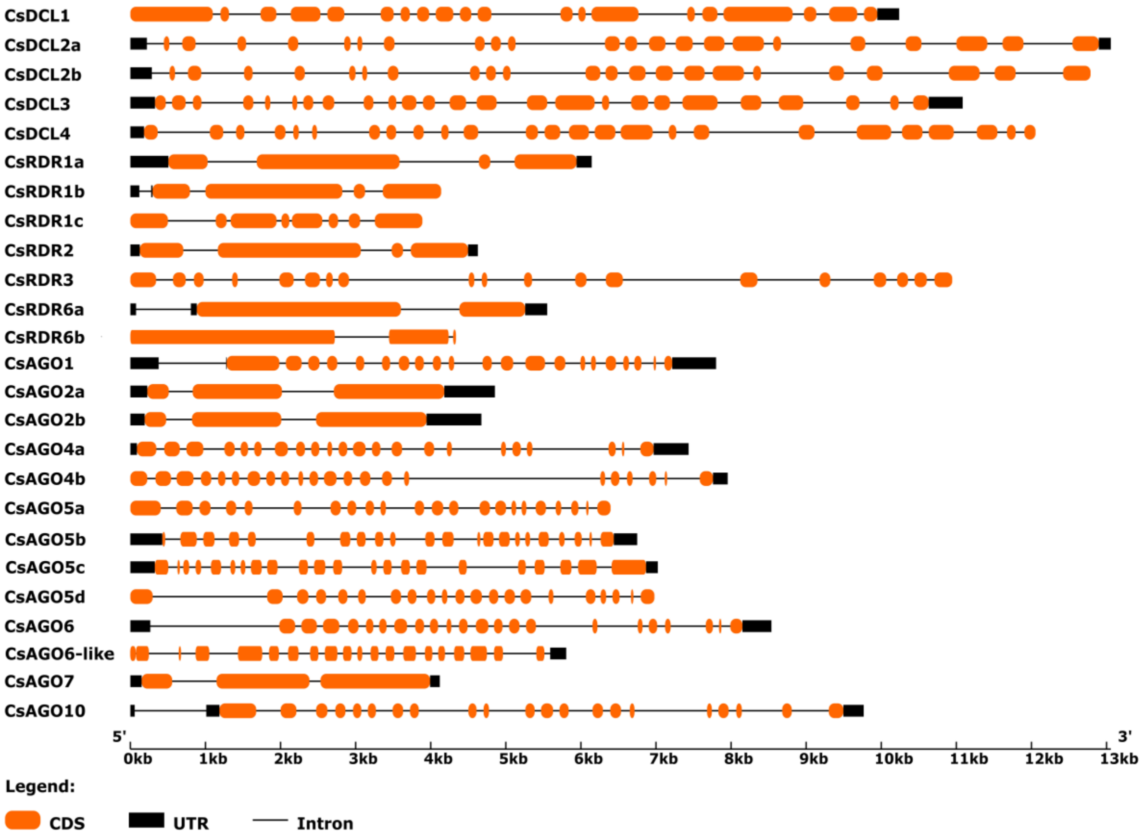

Updated sequences

CsRDR6b: Cs1g14730 - Chromosome 1: from 18.022.840 to 18.027.238

>CsRDR6b
MEPEEREKISVDTQVSVGGFDRDIKAVDLVKYLENEVGGVWRCRLKTSCTPSESYPNFFI
ENVAEVQKTDDYEKVEPHAFVHFATSTYITRAMDAAEHMELFLNGRALKVSLGPENPFRL
NQRGRTSAPLKLPDVYVEIGSLVTQDEFFVSWRGPASGTDFLVDPFDGTCKFCFTKDTAF
ALKSSAEHAVIKCEFKVFLVKEIDIVKQYSELPRVAILLRLASSPRICYRTAEDDIEVLV
PFDLLDDDDPWIRTTDFSPSGAIGRCNSYMLNEVMSYLRKQRVQEDCLSQPVTIREEPDF
EVPMEDPFFCMHYKEGVSFEIMFLVNAVMHKGILNQHQLSDSFFDLLRCQPREVNVTALK
HIYSYKRPVFDAYEKLKDVQEWLIKNPKLYEGPKNIDDIAEVRRLVITPTKAYCLPPEVE
LSNRVLREMTLLKSEMDEGLQTMNANVLSYYVAPIVKDITSNTFSQKTGVFKRVKSILSD
GFNLCGRRYSFLAFSSNQLRDHSAWFFSKVGETSVLDIKNWMGEFTAKNIAKYAARMGLC
FSSTHATVEIPPTEVDHELPDIKRNGYVFSDGIGKITPDLAMEVAQILKLDGSPPPSAYQ
IRYAGYKGVVACWPAKDDGIRMSLRGSMKKFKSCHTSLEICSWTRFQPGFLNRQIITLLS
TLNVSDEIFWTVQASMASKLNLMLVASDVAFEVLTASCAEQGNTAAIMLSAGFKPQTEPH
LQGMLTCIRAAQLWGLRDKARIYVPPGRWLMGCLDELGELKEGQCFIQVSESSLENCFSK
HGSRFAETKKLQVINGFVVIAKNPCLHPGDVRILEAVDRPELHHLCDCLVFPQKGDRPHT
NEASESDLDGDLYFVTWDENLIPPSKKSCPPMEYTPAEAKPLTCHVNHQNLGQICSAHVV
LSEYGAFDENCILLAKLAATAVDSPKTGKIVTMPSHLKPKIYPDFMEKEEYQTYKSNKIL
GRLYRQIKDAYDENISVSSEIDINPVDIHCDTDLEVPESVDFTADARNQKCSYDGQLNGL
LGQYNVNRKEFRHIWSMPKYNSRKQGELQERLKHSYSAFRKEFRQVFEKMNSDFELSDNE

KNVLYEQKASAWYQVTYHREWVKKSLDLLEQGSARSVVLLSFAWIANDYLARIKIKCQEM
ANIGKSKPVNNLARLLIGCKPLCHVK

CsAGO5b: Cs7g17970 - Chromosome 7: from 13.774.473 to 13.781.129

>CsAGO5b
MSRRGGGGRRPDSRRDQPTQAPAPSFQRGGGGAARPRGRGRQGQRGGAGRGSHSGSGAAPSSPH
TASTSTEPAPSSPSVSASASASSSSSVSTLVEETEQKLTLAAPAAATLPPSSSLAVRLPVRPGF
GTVGRKCVVRANHFMVQLAEKDIHHYDVSITPWVTSKKINRQIISQLINLYRLTDLGGRIPAYD
GMKSIYTAGPLPFQSKEFIIELPDSDPRPSSSTRPIRERQFRVVIRLASKPDLYTLQQFLGRRH
FEAPYDVIQVLGVILSAASSEKHTVVGRSFFPTDHGPIGQLGDGVEYWRGYFQSLRLTQMGLSL
NIDVSARSFYEPILVTEFVQNYCRNLSRPLSDQVRLKVKKELKGIKVVLTHLETSNSHRITGIS
SQPMSQLAFTDGSATSMSVIQYFRERYNIALQFTSLPALLAGSEARPIYLPMELSRIVAGQRYT
QRLNERQVTALLQATCQRPREREDYIRMMARANAYNEDTLVNKEFGIQVADDLTSVDARILPAP
MLKYHETGQEASVNPGFGQWNMINKKMFNGGRVEVWTCVNFSTRLNRDVPFQFCQGLVDMCNSK
GMVFNPQPVIPISSSNPNQIEKALVDVHNRTTQQGKQLQLLIIIILPDVSGSYVPRFLVKIDRFA
SLVGGRNTVLVDAVQKRIPLVTDRPTIIFGADVTHPQPWGGTSPSIAAVVASMDWPEVAKYRGL
VSAQAPHEEIIQDLYKSIQDPQWGLVHGGMIRELLIAFRRSTNFKPHRIIFYRDGVGERQFSQV
LLHEMNAIRQACASLEEGYAPPVTFVVVQKRCRTRLFPAEHNRCDLTDRSGNILPGTVVDTEIC
HPTEFDFYLNSHAGIQGTSRPTRYHVLYDENRFTADGLQVLTNNLCYTYARCTRSVSIVPPAYY
AYLAAFRARYYIEDETSAGGSTDGNRSTAERNLAIRPLPVIKDNVKDVMFYC

CsAGO5c: Cs7g17930 - Chromosome 7: from 13.732.122 to 13.738.824

>CsAGO5c
MSRRGGGGRRRDSCRDQPTQAPAPSFQRGGGGAAGPRGRGRQGQRGGAGRGSHSGSGAAP
SSPHTASTSTAPAPSSPSVSESSPSSSSVSTLVEETEQKLSLAAPAAATLPPLSSQELRL
PVRPGIGTVGRKCVVRANHFMVQLAERDIHHYDVSIDQVQDLESNRYSEVTSKKINRQII
SHLINLYGLTNLGGRIPAYDGMKNIYTAGPLPFESKEFIINLPDSDPHPSSSTSPGREGQ
FRVVIRLASMPDLYTLQQFLRRMHFEAPYHVIQVLDVVLRAAPSEKHTVLGRSFFSADLG
PMGQLGDDVEYWRGYFQSLRPTQMGLSLNIDVSASSFYKPILVTEFVQYYCSDLSRPLSD
QVRLKVKKALKGIKVVLRHMGYNLNCKITGITSQPMSQVMFTDGSVTEMSVVQYFLERHN
IALQFTSLPALEAGTEERRIYLPMELSRIVEGQRYTKRLNERQVTALLRATCQRPRDREA
NIQTMARKNAYNKDTLVNKEFGIQVADGLTSFDARILPAPMVIMCHFVLFVTGLWCFQSP
CALKYHESGREASVNPDFGQWNMINKKMFNGGTVQVWTCMNFSTCLNQDVSRFCQRLVDM
CKKKGMVWVFNPQPVIPISSYNPNQIEKELVDVHSKTTQPGKQLQLLIIIILPDVSRSYGE
GRIKRVCETELGIVSQCCQPKHASSRNMQYFENVALKINVKVGGRNTVLVDAVQKRIPLV
TDRPTIIFGADVTHPQPGEDSSPSIAAVVASMDWPEVTKYRGLVSSQAHNEEIIQDLYKS
IKDPQRGLVHGGMIRELLIDFRRSTNFKPHRIIFYRRDGVSEGQFSQVLLHEMNAIRQAC
ASIEEGYAPPVTFVVVQKRHHTRLFPAEYNRRDLTDRSGNILPGTVVDTQICHPTEFDFY
LNSHAGIQQITCVTRNMFTFSLIGFNIALLKNLSVCKVYSISFNRMAVPPAYYAHLAAFR
ARFYIEDETSAGGSTGGTRSTAEAGSLAIRPLPVIKDNVKDVMFYC

CsAGO6-like: Cs6g16080 - Chromosome 6: from 17.010.270 to 17.016.001

>CsAGO6-like
MKFPKDSEAHGPSPSEIPPNVESSQIKSVTGPEVTKKIKPSGSKGEITSLLSNHFKVSIT
GASGHIFHYSVALFYDDGCPIETKGIRRKIIDKVCETYSADLAEMLKESKRTSKLGPTGE
KSLFTISALPHKKMEFLVLLDNPSSYRTTSNDSPDGHGSNNERDRKRRRVSQSKTFKVEI
SVAAKIPLQAIAAALHGQESQNSREAFRVLDIILRQHAAKHGCFLVRQSFFQNEPRSFFD
LGGGVLGCWGFHSSFQATQGGLSLNITTTIIKPGPIIDFLIANQNVRDCYQLDWAKAKRT
LKILRIRVHPFNREYRITGLSDSTCKRQMFSWKSGVKDRNDDVKCVDVTVFDYFVNHRRI
NLCFSGDFPCIDVGKPRKPTYIPIELCSLLSLQRYTKALTVFQRSALVEKSQQKPQEKMK
IITDVMRSNKNDSEPMLRSCGISINSRFAQVEGRILSAPRLMAGNREVIFPKNGRWSFHN
KVIFVPAAKIDHWAVANFSGGCDIRSLCRDLIRFGEMKRISTSPPLNVFEENPQFRRAPA
PVRVDRMFEQMKQKFEKRPCFLLCLLPDRKDSDLYGSWKRKTLSEFGIFNQCLAPTKVNE
QYLMNVLLKINAKLGGLNSLLAIEQSKNLPLVSKVPTIIFGMDVSHGSPGHSNVPSVAAV
VSSRNWPILSRYRASVRSQSTKLEMIDSLFKPLPNKDDAGIVRELLVDFYKSSGQTKPSQ
IIIFRSTLTWRFFFLFIFYAIIAFGTFLFSNIQERCYHLSWIGSKIWKAGSVGLMTSHGK
PVYSLFLLFPRAVSRNSMTLGIKDCGIWLLLGYIRLPETAKTFSYHHNSLPETVKTFNYY
HNSSLNLMSIFAQVSIGLPPYARSDCLWMYQRSTTAVSVVAPVRYAHLAAAQFSQFMKFD
DLSEISSSPGGQTSSGHAHVPALPKLHENVRSSMFFC
